# Alzheimer's disease diagnosis by blood plasma molecular fluorescence spectroscopy (EEM)

**DOI:** 10.1038/s41598-022-20611-y

**Published:** 2022-09-28

**Authors:** Ricardo Fernandes dos Santos, Maria Paraskevaidi, David M. A. Mann, David Allsop, Marfran C. D. Santos, Camilo L. M. Morais, Kássio M. G. Lima

**Affiliations:** 1 Biological Chemistry and Chemometrics, Institute of Chemistry, Federal University of Rio Grande do Norte, Natal, 59072-970 Brazil; 2 Federal Institute of Education, Science and Technology of Rio Grande do Norte-Campus Ipanguaçu, Ipanguaçu, 59508-000 Brazil; 3 Department of Metabolism, Digestion and Reproduction, Imperial College London, London, SW7 2BX UK; 4 Division of Neuroscience & Experimental Psychology, Faculty of Biology, Medicine and Health, School of Biological Sciences, The University of Manchester, Salford Royal Hospital, Salford, UK; 5 Division of Biomedical and Life Sciences, Faculty of Health and Medicine, Lancaster University, Lancaster, UK

**Keywords:** Learning and memory, Fluorescent probes, Medical and clinical diagnostics

## Abstract

Despite tremendous research advances in detecting Alzheimer's disease (AD), traditional diagnostic tests remain expensive, time-consuming or invasive. The search for a low-cost, rapid, and minimally invasive test has marked a new era of research and technological developments toward establishing blood-based AD biomarkers. The current study has employed excitation-emission matrices (EEM) of fluorescence spectroscopy combined with machine learning to diagnose AD using blood plasma samples from 230 individuals (83 AD patients from 147 healthy controls). To evaluate the performance of the classification algorithms, we calculated the commonly used figures of merit (accuracy, sensitivity and specificity) and figures of merit that take into account the samples unbalance and the discrimination power of the models, as F_2_-score (F_2_), Matthews correlation coefficient (MCC) and test effectiveness ($\delta$). The classification models achieved satisfactory results: Parallel Factor Analysis with Quadratic Discriminant Analysis (PARAFAC-QDA) with 83.33% sensitivity, 100% specificity, 86.21% F_2_; and Tucker3-QDA with 91.67% sensitivity, 95.45% specificity and 91.67% F_2_. In addition, the classifiers show high overall performance with 94.12% accuracy and 0.87 MCC. Regarding the discrimination power between healthy and AD patients, the classification algorithms showed high effectiveness with the mean scores separated by three or more standard deviations. The PARAFAC's spectral profiles and the wavelength values from both models loading profiles can be used in future research to relate this information to plasma AD biomarkers. Our results point to a rapid, low-cost and minimally invasive blood-based method for AD diagnosis.

## Introduction

Dementia is the 7th leading cause of death in the world, with Alzheimer's disease (AD) accounting for more than 60% of the cases^1,2^. The AD's main features consist of an insidious onset, slow and progressive loss of memory, alterations in abstract thinking, judgment, behavior, emotions and interference with physical control over the body^1,2^. These AD manifestations have been mainly attributed to the aggregation of amyloid-beta (Aβ) (plaques) and tau proteins (neurofibrillary tangles), as well as to a decrease in neuronal cells^3,4^. Moreover, other physiological changes have also been associated with AD, such as oxidative stress caused by mitochondrial dysfunction, neuroinflammation, and lipid dysregulation^3,5–8^.

Tests performed for the AD diagnosis are generally invasive, such as those requiring lumbar puncture for cerebrospinal fluid (CFS) collection to measure the amyloid-β, tau protein (T-tau) and phosphorized tau protein (P-tau) levels; or are expensive and time-consuming, such as structural neuroimaging techniques (MRI magnetic resonance imaging and computed tomography), positron emission (PET) imaging of cerebral amyloid, or inflamed proteins and neuropsychological tests^9,10^. Despite the large number of tests that can be used for a possible diagnosis of AD, a definitive final diagnosis can still only be provided post-mortem^10,11^. Another strand of studies has worked with less invasive methodologies through biofluids that have shown the emergence of promising biomarkers for diagnosing AD^12^. The use of blood-based biomarkers demonstrates that identification of biochemical changes in the blood could be helpful in the identification of Alzheimer's Disease^10,12–16^. The main issue is that most of these studies provide an isolated analysis of these biomarkers, and they have failed to identify a single biomarker specific to AD^15,17–21^. However, recent studies show that blood-based biomarker panels could be more effective in diagnosing Alzheimer's disease by simultaneously analyzing a number of biochemical changes^14,22–26^.

The identification of biochemical changes and AD diagnosis can be facilitated by using biospectroscopic techniques with applied chemometrics, which can generate spectral fingerprints, giving a more holistic approach to many molecules at the same time and creating models to differentiate between healthy individuals and patients with AD. Infrared (IR) and Raman spectroscopy with chemometrics have been previously used for classification and AD diagnosis by analyzing blood serum or plasma^10,27–31^. However, in relation to molecular fluorescence spectroscopy in excitation and emission matrix (EEM) with applied chemometrics, there are still no studies in the literature, which makes this the first work using EEM for AD diagnosis. The application of fluorescence methods to organic molecules and biochemical problems is remarkable due to the high sensitivity and selectivity of the technique^32^. In addition, there are many biologically important compounds that fluoresce and can be determined, such as amino acids, proteins, coenzymes, vitamins, nucleic acids and many metabolites^32^.

Fluorescence spectroscopy is being widely used as a diagnostic tool in several medical fields, such as mycology, virology, cardiology and oncology^33–37^. Molecular fluorescence spectroscopy is a non-destructive technique^38^ while also being a very sensitive and selective technique when compared to other molecular spectroscopic techniques exploring absorption and scattering phenomena, like IR and Raman, respectively^39^. At the molecular level, it can be used as a tool for detecting interactions in complex sample matrices, such as changes in the function, morphology and microenvironment of cells and tissues^40–43^. The EEM data are obtained from independent excitation and emission profiles for each sample. These data are second-order and can be arranged in a three-way array or cube. Through EEM fluorescence, it is possible to directly monitor the fluorophores present in the sample due to the large amount of analytic information contained in the matrix combined with the high sensitivity of the technique^35,44,45^. Furthermore, the use of 2nd order algorithms with EEM data improves the ability to resolve overlapping spectra allowing the identification of biomolecules^35,46,47^.

The large number of overlapping spectrums in the EEM fluorescence technique, and even quenching effects, due to the sample matrix complexity can lead to moderate selectivity^47,48^. To account for this issue and extract as much relevant information as possible from EEM fluorescence, chemometric methods of 2nd-order can be used^48^. PARAFAC is the most widely used 2nd-order algorithm in studies with EEM fluorescence^49^.

In this work, we show a new approach to diagnosing AD in blood plasma using EEM fluorescence spectroscopy and 2nd-order algorithms. In this pioneering work, we evaluated the performance of classification models with figures of merit that consider the sample unbalance and discriminatory power of the models and defined the diagnostic accuracy, sensitivity and specificity of the technique. Our results might contribute to a new screening tool for the non-invasive and cheaper diagnosis of Alzheimer's disease in people with suspected disease.

## Materials and methods

**Figure 1:**
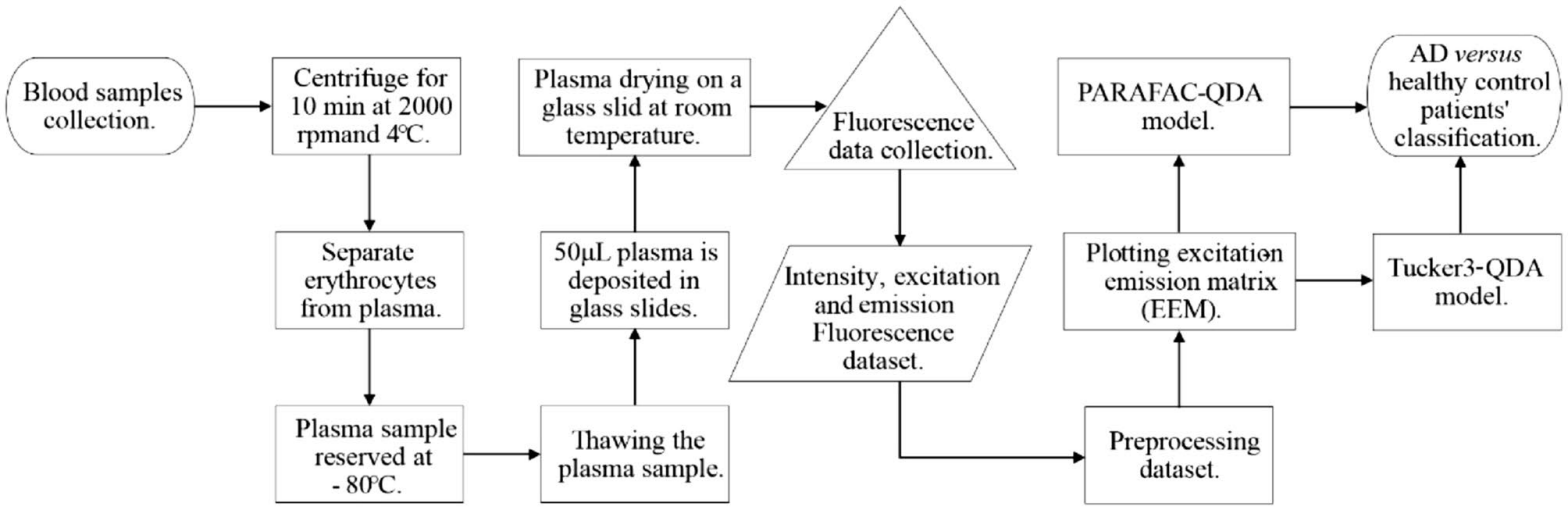
Workflow for the AD versus healthy control classification.

The methodology used in this study is summarized in the workflow in Fig. 1.

### Patient groups

This study's cohort was divided into two classes: 83 patients with AD and 147 healthy volunteers. All patients diagnosed with AD underwent psychological examinations (Manchester Neuropsychology Inventory) performed at the Brain Function Unit at the Greater Manchester Neurosciences Centre, Salford Royal Hospital. All methods were performed in accordance with the relevant guidelines and regulations. The control group was comprised of close relatives who escorted patients in their medical appointments.

### Plasma collection and preparation

Blood samples were collected at Salford Royal Hospital, according to Local Ethical Approval (05/Q1405/24 conferred by North West 10 Research Ethics Committee Greater Manchester North). The informed consent was obtained from all the voluntaries. Plasma was obtained from blood samples collected in EDTA tubes and centrifuged for 10 min at 2000 rpm and 4 °C to separate erythrocytes from plasma. Samples were aliquoted into new microtubes and reserved at − 80 °C. Before spectroscopic analysis, plasma samples were thawed and 50 μL of each sample was deposited in glass slides (MirrIR Low-E slides; Kevley Technologies), which were left to dry at room temperature.

### EEM fluorescence spectroscopy

To obtain the EEM matrices, the RF-5301 Shimadzu spectrofluorometer operated by RFPC Software was used. The sample area was adapted with a base for fitting the glass blades. The wavelength range worked for excitation was 250–350 nm with intervals of 10 nm, while for emission was 300–850 nm with intervals of 1 nm. Each analysis was configured with super mode scanning speed (3000 nm/min), high sensitivity and spectral bandwidth of 3 nm for excitation and emission.

### Spectral preprocessing

Spectral data preprocessing was performed with MATLAB R2014b Software (The Math-Works, Natick, USA) with the implementation of the PLS Toolbox 7.9.3 package (Eigenvector Research Inc., USA). First, using the 'EEMscat'1 algorithm, Rayleigh and Raman's scatterings were removed. As a second step, a multiplicative signal correction (MSC) was performed to correct the additive and multiplicative effects of light scattering caused by physical phenomena.

### Chemometric analysis

Chemometric analysis of the spectra was performed using MATLAB R2014b Software (The Math-Works, Natick, USA) with MATLAB toolbox TTWD-DA 1.0^50^, used for second-order data classification using discriminant analysis techniques. For model construction, a data cube of dimension 230 × 11 × 551 was generated, composed of the number of samples and by the excitation (250–350 nm with steps of 10 nm) and emission wavelengths (300–850 nm with steps of 1 nm), respectively. The samples were divided into training (*n* = 162), validation (*n* = 34) and tests (*n* = 34) using the Kennard-Stone sample selection algorithm^51^. This division generated three-way arrays with the respective dimensions: 162 × 11 × 551, 34 × 11 × 551 and 34 × 11 × 551. The training samples were used for the construction of the models; the validation samples were used for the internal optimization of the models; and the test samples, to assess the classification models' quality.

The models performed in this study were Tucker3-QDA and PARAFAC-QDA. Tucker3 is a multi-way decomposition method for data organized into three or higher-order arrays^52^. This method is a generalization of PCA (Principal Components Analysis) to higher orders, so it is also known as three-way principal components analysis^52,53^. The structural basis of the Tucker3 model for the deployment of a three-way array **X** can be expressed by the Eq. (1)^52,54,55^:

1 $${{\varvec{x}}}_{{\varvec{i}}{\varvec{j}}{\varvec{k}}\boldsymbol{ }=}\sum_{{\varvec{r}}=1}^{{\varvec{R}}}\sum_{{\varvec{s}}=1}^{{\varvec{S}}}\sum_{{\varvec{t}}=1}^{{\varvec{T}}}{{\varvec{a}}}_{{\varvec{i}}{\varvec{r}}}{{\varvec{b}}}_{{\varvec{j}}{\varvec{s}}}{{\varvec{c}}}_{{\varvec{k}}{\varvec{t}}}{{\varvec{g}}}_{{\varvec{r}}{\varvec{s}}{\varvec{t}}}+\boldsymbol{ }{{\varvec{e}}}_{{\varvec{i}}{\varvec{j}}{\varvec{k}}}\boldsymbol{ }\boldsymbol{ }$$

In Eq. (1), ${{\varvec{x}}}_{{\varvec{i}}{\varvec{j}}{\varvec{k}}}$ represents each element of the three-way array **X**, which is the product of the multiplication of four values (${\varvec{a}}$, ${\varvec{b}}$, ${\varvec{c}}$ and ${\varvec{g}}$) and a small unmodeled part ${{\varvec{e}}}_{{\varvec{i}}{\varvec{j}}{\varvec{k}}}$, that contain the errors of the model. The values of ${\varvec{a}}$ are the Tucker3 model scores and are stored in the matrix **A**, or Tucker3 score matrix, which represents the sample direction. The values of ${\varvec{b}}$ are the loadings and are stored in the matrix **B**, or Tucker3 loading matrix, which represents the 1st mode (e.g., excitation) direction. The values of ${\varvec{c}}$ are also loadings and are stored in the matrix **C**, or Tucker3 loading matrix, which represents the 2nd mode (e.g., emission) direction. The values of ${\varvec{g}}$ are the loadings of interaction between the factors and are stored in the matrix **G**, or the core matrix.

The structural basis for the Tucker3 model can also be written in matrix form^53^:

2 $${\mathbf{\underline {X} }} = {\mathbf{AG}}\left( {{\mathbf{C}} \otimes {\mathbf{B}}} \right)^{{\mathbf{T}}} + {\mathbf{\underline {E} }}$$

where ${\mathbf{\underline {E} }}$ is a residual three-way array and $\otimes$ represents the Kronecker product^56^.

The other multi-way decomposition method for higher-order data used in our study was PARAFAC. This method is based on the possibility that simultaneous factor analysis of distinct matrices in parallel can generate a single ideal set of factors^57^. The structural basis of the PARAFAC model for the decomposition of a three-way array **X** can be expressed by the Eq. (3)^49,58^:

3 $${{\varvec{x}}}_{{\varvec{i}}{\varvec{j}}{\varvec{k}}\boldsymbol{ }=}\sum_{{\varvec{f}}=1}^{{\varvec{F}}}{{\varvec{a}}}_{{\varvec{i}}{\varvec{f}}}{{\varvec{b}}}_{{\varvec{j}}{\varvec{f}}}{{\varvec{c}}}_{{\varvec{k}}{\varvec{f}}}+\boldsymbol{ }{{\varvec{e}}}_{{\varvec{i}}{\varvec{j}}{\varvec{k}}}$$

According to Eq. (3) and considering the case of a fluorescence excitation-emission matrix, ${{\varvec{x}}}_{{\varvec{i}}{\varvec{j}}{\varvec{k}}}$ is the intensity corresponding to the sample ${{\varvec{i}}}{{\rm th}}$ in the variable ${{\varvec{j}}}{{\rm th}}$ (excitation mode) and in the variable ${{\varvec{k}}}{{\rm th}}$ (emission mode). ${\varvec{f}}$ corresponds to the number of components, and each of these components has: $I a$-values (scores), one for each sample and are stored in the matrix **A**, or PARAFAC scores matrix, which represents the sample direction; $J b$-values (loadings), one for each excitation wavelength and are stored in the matrix **B**, or PARAFAC loadings matrix, which represents the 1st mode (excitation) direction; $K c$- values, one for each emission wavelength and are stored in the matrix **C**, or PARAFAC loadings matrix, which represents the 2nd mode (emission) direction.

The PARAFAC model also has its representation of the structural base in matrix form^53^:

4 $${\mathbf{\underline {X} }} = {\mathbf{A}}\left( {{\mathbf{C}}\left| \otimes \right|{\mathbf{B}}} \right)^{{\mathbf{T}}} + {\mathbf{\underline {E} }}$$

where ${\mathbf{\underline {E} }}$ is a residual three-way array; and $\left| \otimes \right|$ represents the Khatri-Rao product^59^.

Tucker3 and PARAFAC perform an intelligent data reduction in the construction of classification models, working as an exploratory data analysis^34^. Thus, after the unfolding performed by these two techniques, the matrix scores from Tucker3 and PARAFAC were inserted as input variables for the QDA algorithm^50^. QDA is a classification algorithm that calculates classification scores using Mahalanobis distance according to the Eq. (5)^35,60^:

5 $${{\varvec{Q}}}_{{\varvec{i}}{\varvec{k}}\boldsymbol{ }}=({{\varvec{x}}}_{{\varvec{i}}}-{\overline{{\varvec{x}}} }_{{\varvec{k}}}{)}^{{\varvec{T}}}{\boldsymbol{\Sigma }}_{{\varvec{k}}}^{{\varvec{T}}}({{\varvec{x}}}_{{\varvec{i}}}-{\overline{{\varvec{x}}} }_{{\varvec{k}}}{)}^{{\varvec{T}}}+\boldsymbol{ }{{\varvec{l}}{\varvec{o}}{\varvec{g}}}_{{\varvec{e}}}\left|{{\varvec{\Sigma}}}_{{\varvec{k}}}\right|-2{{\varvec{l}}{\varvec{o}}{\varvec{g}}}_{{\varvec{e}}}{{\varvec{\pi}}}_{{\varvec{k}}}$$

where ${{\varvec{Q}}}_{{\varvec{i}}{\varvec{k}}}$ is the classification score of the sample ${{\varvec{i}}}$ of class ${\varvec{k}}$; ${{\varvec{x}}}_{{\varvec{i}}}$ is the vector containing the classification variables for the sample ${\varvec{i}}$ (e.g., scores from Tucker3 or PARAFAC); ${\overline{{\varvec{x}}} }_{{\varvec{k}}}$ is the average vector of class ${\varvec{k}}$; ${{\varvec{\Sigma}}}_{{\varvec{k}}}$ is the variance–covariance matrix of class ${\varvec{k}}$; ${{\varvec{\pi}}}_{{\varvec{k}}}$ is the prior probability of class k. Equations (6) and (7) show how ${{\varvec{\Sigma}}}_{{\varvec{k}}}$ and ${{\varvec{\pi}}}_{{\varvec{k}}}$ are calculated:

6 $${{\varvec{\Sigma}}}_{{\varvec{k}}}=\frac{1}{{{\varvec{n}}}_{{\varvec{k}}}-1}\sum_{{\varvec{i}}=1}^{{{\varvec{n}}}_{{\varvec{k}}}}\left({{\varvec{x}}}_{{\varvec{i}}}-{\overline{{\varvec{x}}} }_{{\varvec{k}}}\right)({{\varvec{x}}}_{{\varvec{i}}}-{\overline{{\varvec{x}}} }_{{\varvec{k}}}{)}^{{\varvec{T}}}$$

7 $${{\varvec{\pi}}}_{{\varvec{k}}}=\frac{{{\varvec{n}}}_{{\varvec{k}}}}{{\varvec{n}}}$$

where ${\varvec{n}}$ is the number of total objects in the training set; ${\varvec{c}}$ is the number of classes; and ${{\varvec{n}}}_{{\varvec{k}}}$ is the number of objects of class ${\varvec{k}}$.

### Model quality assessment

The evaluation of the quality of the models was carried out through the calculation of different figures of merit based on the errors and successes of prediction of the classes, in which TP, TN, FP and FN mean true positive, true negative, false positive and false negative, respectively. The calculated figures of merit were: correction classification rate (CC%. Equation 8); accuracy (AC, Eq. 9); sensitivity or recall (SENS, Eq. 10); specificity (SPEC, Eq. 11); Matthews Correlation Coefficient (MCC, Eq. 12); F_2_-score (Eq. 13); precision (Eq. 14); Youden's index ($\gamma$, Eq. 15); positive and negative likelihoods (*ρ*_+_ and *ρ*_*−*_, Eqs. 16 and 17) and test effectiveness ($\delta$, Eq. 18). The figures of merit are described below^34,61–63^:

8 $$CC\%=100-\frac{\left({\varepsilon }_{1}-{\varepsilon }_{2}\right)}{N} \times 100$$

9 $${AC}_{\%}=\frac{(TP+TN)}{TP+TN+FP+FN} \times 100$$

10 $${SENS}_{\%}=\frac{TP}{TP+FN} \times 100$$

11 $${SPEC}_{\%}=\frac{TN}{TN+FP} \times 100$$

12 $$MCC=\frac{TP.TN-FP.FN}{\sqrt{\left(TP+FP\right) \cdot \left(TP+FN\right) \cdot \left(TN+FP\right).(TN+FN)}}$$

13 $${F}_{\beta }= \left(1+{\beta }^{2}\right) \cdot \frac{{P}_{\%} \cdot {SENS}_{\%}}{{{\beta }^{2} \cdot P}_{\%}+{SENS}_{\%}}$$

14 $${P}_{\%}= \frac{TP}{TP+FP} \times 100$$

15 $$\gamma = SENS-(1-SPEC)$$

16 $${\rho }_{+}= \frac{SENS}{1-SPEC}$$

17 $${\rho }_{-}= \frac{1- SENS}{SPEC}$$

18 $$\delta = \frac{\sqrt{3}}{\pi }\left[\mathrm{ln}\left(\frac{SENS}{1-SENS}\right)+\mathrm{ln}\left(\frac{SPEC}{1-SPEC}\right)\right]$$

In Eq. (8), $CC\%$ corresponds to the percentage of samples correctly classified considering their true classes in each step (training, validation and testing). A binary approach is used to calculate $CC\%$, where ${\varepsilon }_{1}$ means the errors for class 1 and ${\varepsilon }_{2}$ the errors for class 2; $AC$ indicates the number of correct answers of the model in relation to all classes; $SENS$ expresses the percentage of positives correctly pointed out by the model; $SPEC$ expresses the percentage of negatives correctly pointed out by the model; $MCC$ is the correlation coefficient between the actual values and the predicted values, it is not affected by the data unbalance; ${F}_{\beta }$ is a happy medium between sensitivity and precision, which can express an overall classification performance as long as the parameter $\beta$ is used correctly; $P$ is the percentage of relevant correct answers in relation to all predictions made by the model for a given class; $\gamma$ evaluates the ability of models to avoid failures; ${\rho }_{+}$ and ${\rho }_{-}$ provide an assessment of the model's performance in relation to positive and negative class, respectively; $\delta$ estimates how successful the model is able to differentiate the positive from the negative class, is a measure of the discriminatory power of the model.

## Results

**Figure 2:**
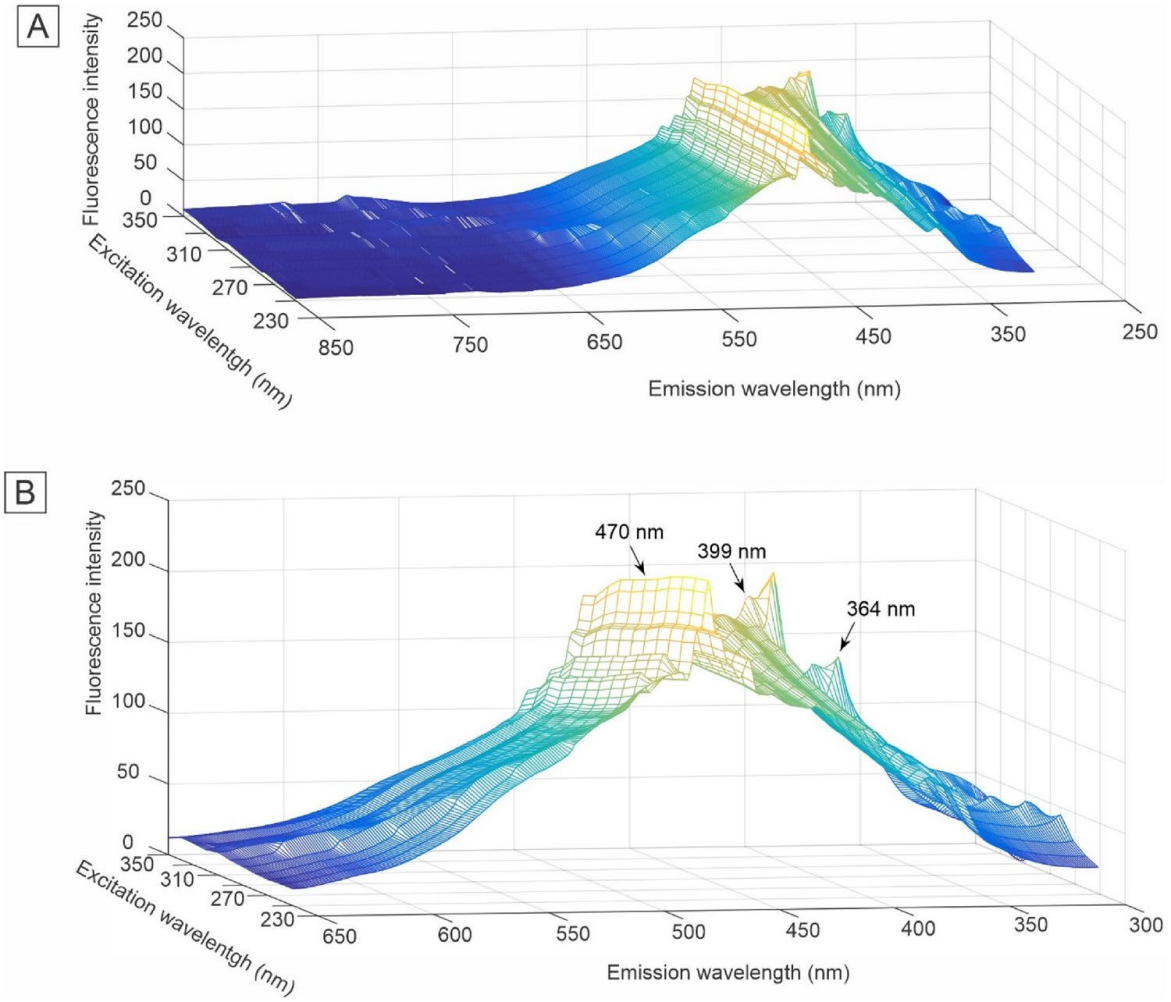
Preprocessed healthy control group excitation-emission matrix (EEM) molecular fluorescence spectrums for blood plasma samples: (**A**) all spectral region investigated and (**B**) emphasis in the spectral region of the most relevant peaks.

**Figure 3:**
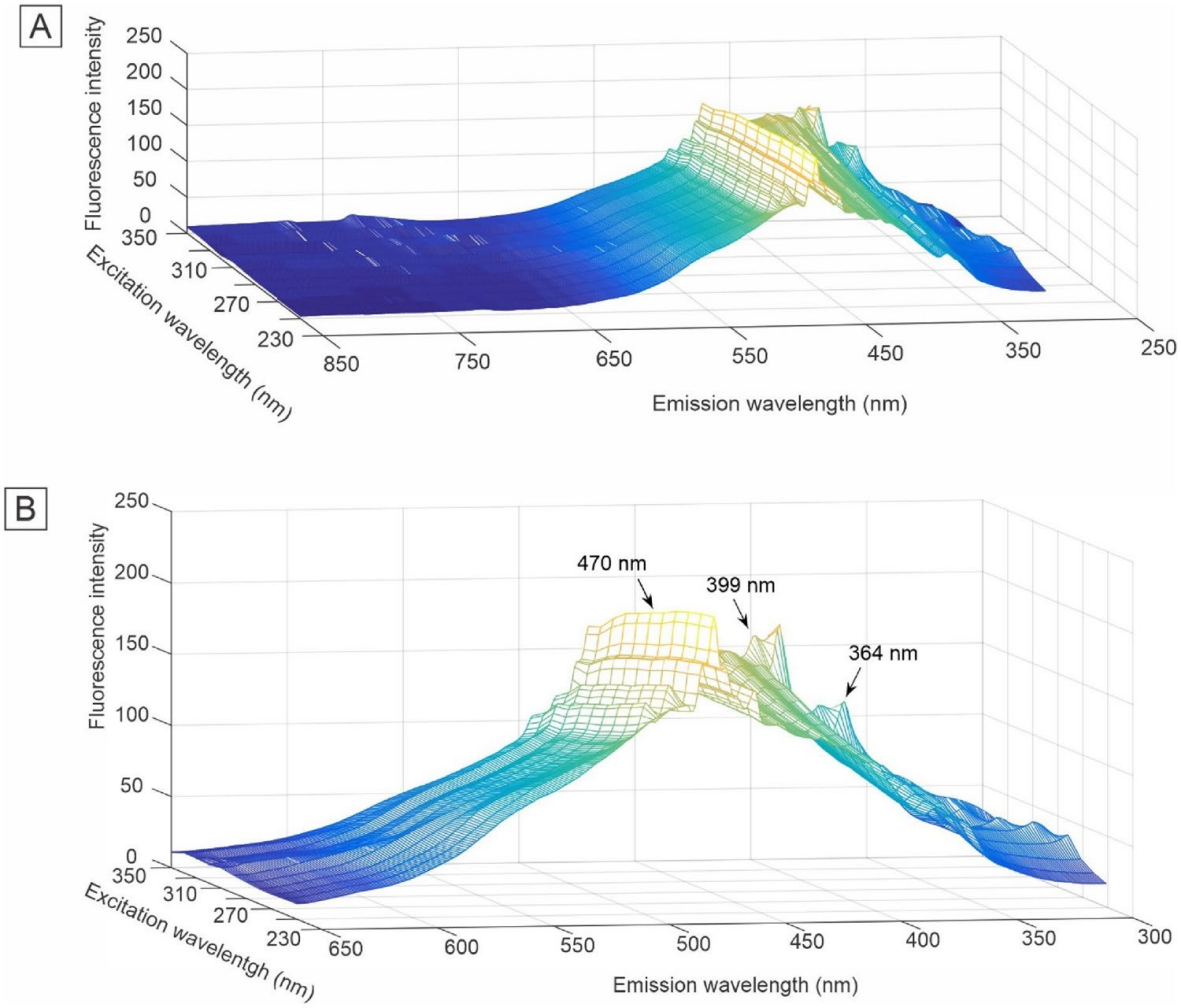
Preprocessed Alzheimer disease group excitation-emission matrix (EEM) molecular fluorescence spectrums for blood plasma samples: (**A**) all spectral region investigated and (**B**) emphasis in the spectral region of the most relevant peaks.

The fluorescence dataset collected from plasma samples collected from controls and AD patients were plotted in the excitation-emission matrix (EEM) (Figs. 2 and 3). The spectra of each class were preprocessed with multiplicative scattering correction (MSC) and removal Rayleigh and Raman scattering (Figs. 2 and 3). In this way, the 2nd-order exploratory analysis algorithms PARAFAC and Tucker3 were used, followed by the supervised classification technique Quadratic Discriminant Analysis (QDA) to build classification models. These models can more accurately evidence the differences between the different classes.

### PARAFAC-QDA

**Figure 4:**
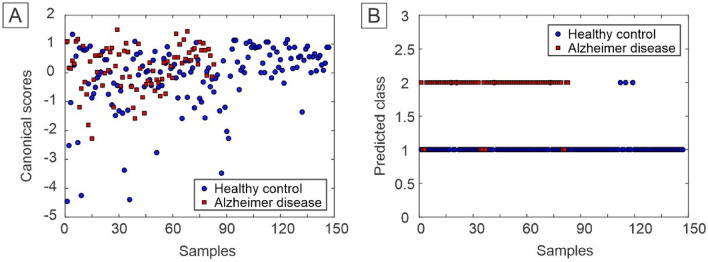
(**A**) Canonical scores of the PARAFAC and (**B**) predicted class values by PARAFAC-QDA.

For the construction of the PARAFAC-QDA model, 6 factors were selected, which covered 96.31% of the explained variance of the data. Table 1 shows the correct classification rates (CC%) for the models used in this study. Both models presented the best CC% for the control class, except for the PARAFAC-QDA validation set that obtained a value of 100% for AD. In the case of the PARAFAC-QDA model, the value of 100% for the healthy control test set can also be highlighted. Canonical scores for the PARAFAC and the predict class values for the PARAFAC-QDA model are represented in Fig. 4A,B, respectively. Through the predict class values, it was possible to verify a good classification between the groups of samples, which was not possible to observe only with the canonical scores.

**Table 1:** Correct classification rate for PARAFAC-QDA and Tucker3-QDA models.

Models	Correct classification rate	Alzheimer's disease	Healthy control
PARAFAC-QDA	Training (%)	91.52	96.12
	Validation (%)	100	86.36
	Test (%)	83.33	100
Tucker3-QDA	Training (%)	94.91	97.09
	Validation (%)	75	90.91
	Test (%)	91.67	95.45

To assess the quality of the built models, figures of merit were calculated (Table 2). The PARAFAC-QDA model was able to classify samples between healthy control and AD with 100% specificity and precision, with all other metrics above 83% and MCC of 0.87. Due to the similarity between the MCC values for both models, and for a comparison between the quality of the discriminants, the test effectiveness and positive and negative likehoods values were calculated, which were higher for the PARAFAC-QDA model. The scores (Fig. 6A), excitation (Fig. 6B) and emission loadings (Fig. 6C) for the first three factors used in the PARAFAC-QDA model are shown in Fig. 6. The scores profile of the PARAFAC (Fig. 6A) shows overlapping AD and control samples without the possibility of separating the classes. The most important wavelength values for model classification are 250 nm, 260 nm and 340 nm for excitation (Fig. 6B); and 399 nm and 470 nm for emission (Fig. 6C).

**Table 2:** Figures of merit for PARAFAC-QDA and Tucker3-QDA models.

Figures of merit	Models	
	PARAFAC-QDA	Tucker3-QDA
Accuracy (%)	94.12	94.12
Sensitivity (%)	83.33	91.67
Specificity (%)	100	95.45
Precision (%)	100.00	91.67
F_2_-score (%)	86.21	91.67
MCC	0.87	0.87
${\varvec{\gamma}}$ (%)	83.33	87.12
${{\varvec{\rho}}}_{+}$	∞	20.17
${{\varvec{\rho}}}_{-}$	0.17	0.087
${\varvec{\delta}}$	∞	3.00

PARAFAC-QDA: parallel factor analysis-quadratic discriminant analysis; Tucker3-QDA: Tucker3-quadratic discriminant analysis; MCC: Matthews correlation coefficient; ${\varvec{\gamma}}$: Youden's index; ${\rho }_{+}$ and ${\rho }_{-}$: likehoods ratios; $\delta$: test effectiveness.

### Tucker3-QDA

**Figure 5:**
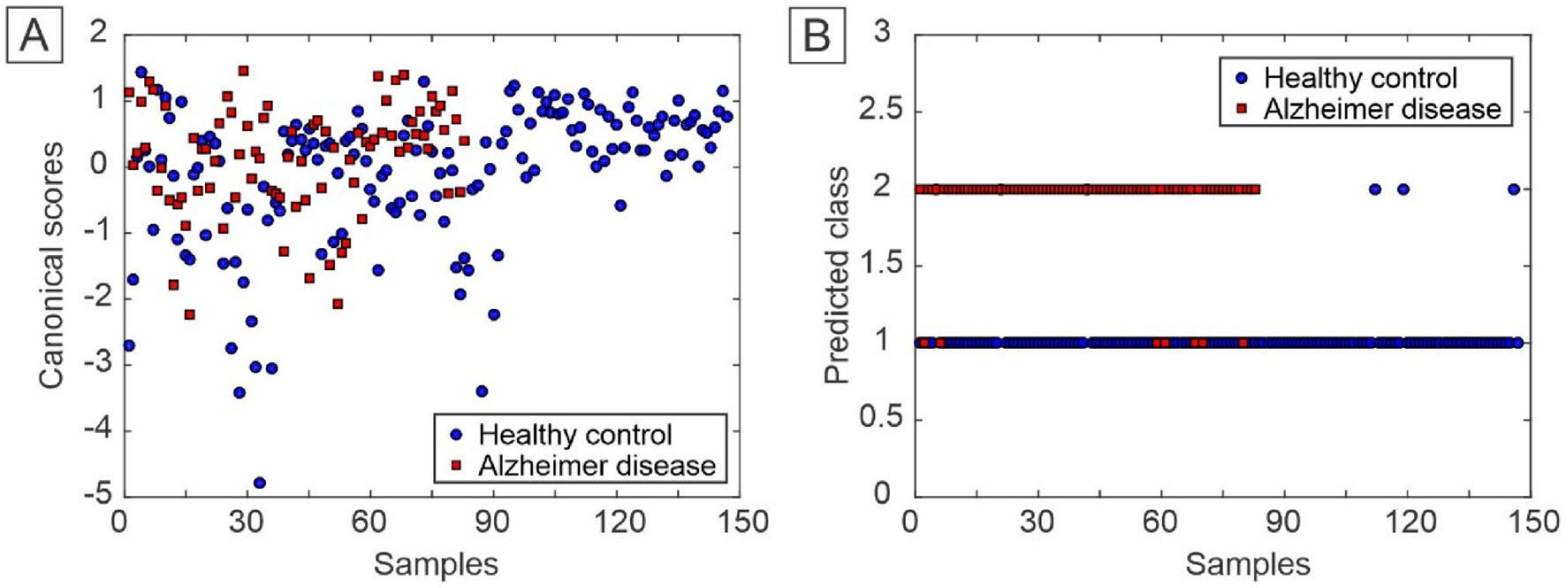
(**A**) Canonical scores of the Tucker3-QDA and (**B**) predicted class values by Tucker3-QDA.

The Tucker3-QDA model was built with 6 factors, which explained 96.73% of the variance of the studied data. Regarding the correct classification rate (CC%), the model presented values mostly higher than those of the PARAFAC-QDA model in all steps (Table 1). Only the values of 75% for AD of CC% in the validation step and 95.45% for the healthy control group in the test step were lower than the PARAFAC-QDA model. Figure 5 presents the canonical scores for Tucker3 (Fig. 5A) and the predict class values for Tucker3-QDA (Fig. 5B). The predict class values showed a good classification for the model, despite the non-segregation of groups in the canonical scores.

The figures of merit for the Tucker3-QDA model presented most values above 90% and MCC of 0.87, like the PARAFAC-QDA model (Table 2). Figure 7 shows the scores (Fig. 7A), excitation (Fig. 7B) and emission loadings (Fig. 7C) for the first three factors used in the construction of the Tucker3-QDA model. Tucker3 scores showed no separation between healthy control and AD classes (Fig. 7A). The excitation and emission loadings indicated as the most important wavelengths the values of 300 nm and 350 nm for excitation (Fig. 7B); and 364 nm and 470 nm for emission (Fig. 7C).

## Discussion

Blood plasma analysis through the EEM fluorescence spectroscopy technique and multivariate classification models showed promising results for the discrimination between healthy controls and patients with Alzheimer's disease. Our diagnostic accuracy results are consistent with previous studies that used blood samples analyzed by spectroscopy or other techniques for AD diagnoses, such as neurofilament light chain (NfL) and lipids or protein-based plasma biomarkers panel^9,10,19,26,27,31^.

Visual inspection of the fluorescence spectral profiles of healthy controls (Fig. 2B) and AD (Fig. 3B) shows a small decrease in fluorescence intensity in the region between approximately 360 and 470 nm. This decrease can be attributed to pathophysiological alterations in the analyzed sample matrix, such as mitochondrial dysfunction, since the region between 360 and 470 nm may correspond to biomolecules such as tryptophan, collagen, elastin, NADH and flavins^64^. In addition to these biomolecules, peaks with lower intensities can be attributed to a variety of vitamins and lipids that can autofluoresce in this region^65^. However, making statements by simple observation of the spectral profiles is inadequate in evaluating these results, as these spectra correspond to an average of the spectra. In this way, the controls and AD spectra present great similarity and overlap if plotted together, which makes it impossible to draw any conclusion only by visual inspection.

The Kennard-Stone (KS) algorithm can reduce the contribution of spectral changes that may affect the classification result. This occurs in the sample selection process in which the algorithm chooses the most distant samples from the centers of their classes to be part of the training set^34^. In this way, the test and validation sets will have samples with smaller changes, but with the changes that mostly concern each class^34^. Cross-validation is another technique that could be used for model optimization, where the training parameters, such as the number of PARAFAC factors, are selected based on the lowest cross-validation error, and then the model is tested in an external test set for final model validation. In machine learning, the use of cross-validation is optional, and it is recommended for small datasets. For bigger datasets, such as those worked in this study, it is preferable to have an external validation set for model optimization. Consequently, the dataset is split into training, validation and test. The model evaluation performed with the validation set is the same as the evaluation for cross-validation (to find the optimum model parameters). However, by having a separated validation set, the samples used for validation are different from the training samples, thus giving a more realistic performance towards the test samples.

The PARAFAC-QDA and Tucker3-QDA showed an excellent model fit, which can be seen in Table 1, in which most of the CC% values are between 83, 33 and 100% for each set of samples in both classes. The Tucker3-QDA model obtained the lowest CC% value, 75%, for the HC class in the validation phase, which did not prevent the excellent fit of the model. Having a fitted model means that the models were able to generate scores that correlated with the characteristics of the samples being able to differentiate them^34^. There was a mix of samples when based only on the factors (Figs. 4A and 5A), proving that when carrying out only the exploratory analyses is not enough to class the samples. However, after applying the classification algorithm (QDA) the correct classification rate increased significantly, as seen in Figs. 4B and 5B.

The accuracy, sensitivity and specificity values obtained for PARAFAC-QDA and Tucker3-QDA, which are presented in Table 2, were high and comparable to studies already carried out using spectroscopic techniques or other blood-based methods for AD diagnosis. More specifically, the IR and Raman techniques have previously achieved values between 86 and 98.4% for these three metrics^10,27,28,30^. Whilst other blood-based molecular methods, like NfL and lipids-based plasma biomarkers panel, achieved 87% and 90% accuracy, respectively^9,19^. Another study with a proteins-based plasma biomarkers panel achieved 85% sensitivity and specificity with an area under the receiver operating characteristic curve (AUC) of 89%^66^. These studies evaluated the effectiveness of the techniques and used models mainly through sensitivity, specificity and accuracy values. The first two figures of merit assess the algorithm's effectiveness with respect to a single class (positive or negative), while accuracy assesses the overall effectiveness of the algorithm through the ratio between the total number of correctly classified samples and the total number of samples^62^. However, to evaluate the unbalanced data of our study, the precision/recall pair may be more informative than the sensitivity/specificity pair^67–69^. For this reason, in addition to these commonly calculated metrics, we also calculated precision, recall and consequently the ${F}_{2}{\text{-}}score$ (Table 2). We chose to calculate the ${F}_{2}{\text{-}}score$, which gives twice as much weight to recall as precision^70^, because if individuals with the disease are misclassified as healthy (false negatives), they do not undergo further tests and thus cannot be identified at an early stage. Emphasizing the recall means giving more importance to the fraction of patients classified as AD who actually have the disease. The Tucker3-QDA model showed a value of ${F}_{2}{\text{-}}score$ higher than PARAFAC-QDA (Table 2), but both values were above 85%, which represents a good result for the diagnosis of AD.

The use of accuracy alone to evaluate the performance of unbalanced models is not considered reliable, as it provides an overvalued estimate of the model's classification ability in relation to the majority class^71–74^. Therefore, for an evaluation of the general performance of the models used, we also calculated the Mathews Correlation Coefficient (MCC), which is not affected by the unbalanced data^61,75^. MCC only generates high results, close to 1, if the model can correctly predict most positive and negative cases^61,76,77^. MCC values close to 1 indicate an excellent classifier, while values close to -1 indicate an erroneous classifier. The classifiers in our study showed similar MCC values of 0.87 (Table 2), which indicates that the two models can be considered good classifiers for both positive (AD) and negative (controls) cases.

As a complement to the metrics that evaluated the effectiveness of the models in general, we calculated the ability of the models to avoid failures through Youden's index. This figure of merit gives similar weight to positive and negative cases, which favors the overall evaluation of the model regarding this metric without favoring just a single class^78^. As can be seen in Table 2, both models presented values above 83% in relation to failure prevention, and the Tucker3-QDA model was the most effective in avoiding errors.

The last calculated figures of merit were likehoods ratios (${\rho }_{+}$ and ${\rho }_{-})$ and test effectiveness ($\delta$). The likehoods ratios were used to compare the performance of classification algorithms with respect to both classes^71,79^. When comparing ${\rho }_{+}$ and ${\rho }_{-}$ values for our models, PARAFAC-QDA obtained the highest values (Table 2). This means that this algorithm is better at confirming positive AD cases, while Tucker3-QDA is better at confirming negative AD cases^71,79^. The test's effectiveness demonstrates how well a classifier can distinguish the positive from the negative class, and it demonstrates the discriminatory power of the model^63^. After comparing the two models, PARAFAC-QDA model presented a higher discriminatory power than the Tucker3-QDA model. Values of δ from 3.0 are considered highly effective and indicate that the mean scores of AD and HC are separated by three standard deviations^63^.

**Figure 6:**
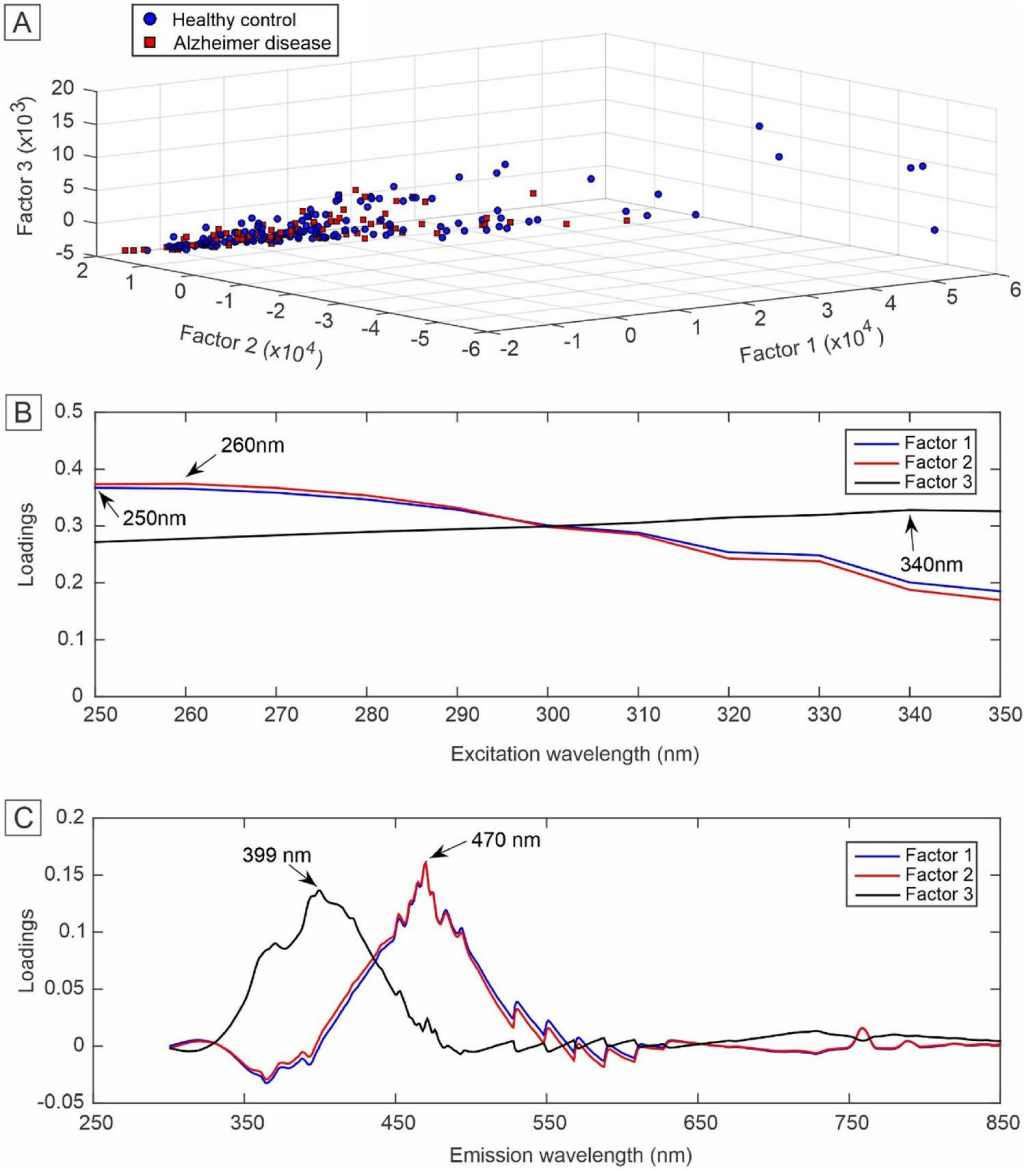
Scores and loadings for the first three factors selected for the PARAFAC. (**A**) Scores; (**B**) loadings for excitation; and (**C**) loadings for emission.

**Figure 7:**
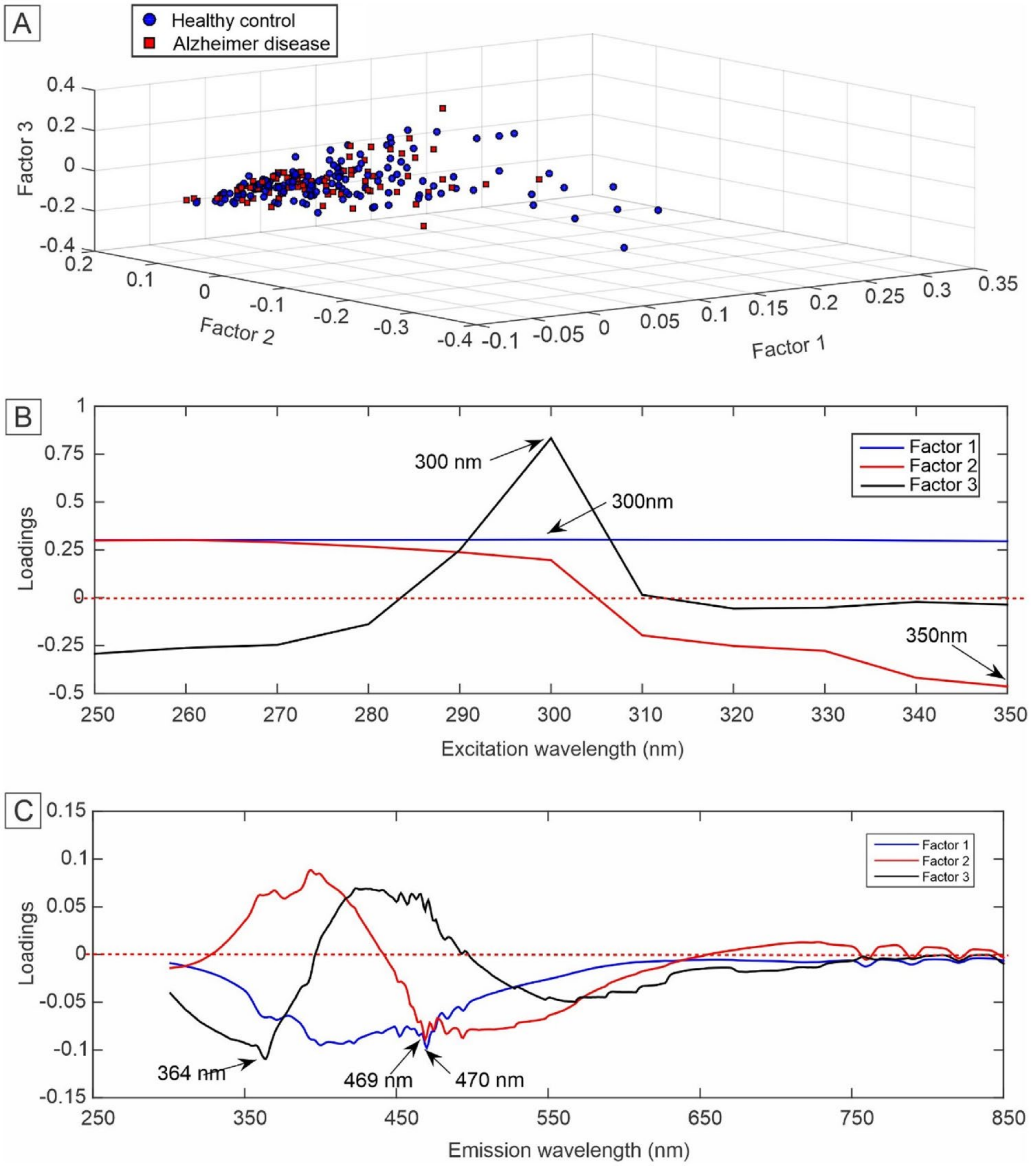
Scores and loadings for the first three factors selected for the Tucker3. (**A**) Scores; (**B**) loadings for excitation; and (**C**) loadings for emission.

Regarding the scores presented in Figs. 6A and 7A, these values are proportional to the concentration of the constituents of the samples, and even if the scale factor between the scores and the real concentrations is not known, they can be interpreted as the relative concentration of the analyte in the sample^34,80^. From the exploratory analysis of the data, through of the plotting of scores as a function of the first three factors for each model (Figs. 6A and 7A), there was no separation of the groups.

The loading plots show the most important excitation (Figs. 6B and 7B) and emission (Figs. 6C and 7C) wavelengths that differentiate the classes used in constructing the PARAFAC-QDA and Tucker3-QDA models. The best explained variances for each classification model were found with six factors. However, as there is a loss of chemical information in loadings when there is a large number of factors, the loading plots were constructed with only the first three factors provided for each model (Figs. 6B,C and 7B,C). Thus, three factors generated three excitation and emission wavelengths for the PARAFAC -QDA as well as for the Tucker3-QDA. The values of the wavelengths present in the loading plots can be comprehended as "biological markers"^34^. Furthermore, in the case of PARAFAC, the emission loadings (Fig. 6C) are recoveries of the spectral profiles of the species that are contributing to the fluorescence signal^80,81^.

The excitation peaks at 250 nm and 260 nm (Fig. 6B) and the emission peaks at 470 nm (Fig. 6C) for the PARAFAC-QDA model can be attributed to a mitochondrial dysfunction commonly associated with AD^64,82–84^. This excitation/emission region of the fluorescence spectrum concerns the intracellular coenzyme NADH, which plays a fundamental role in cellular oxidation–reduction reactions^64^. In the literature, it has already been reported that there are changes in mitochondrial enzymes linked to NADH in the brain of patients with AD^5,85^. The 340 nm/399 nm pair (excitation/emission), also for the PARFAC-QDA model, may be related to the Tyrosine residue (Tyr_10_) present in beta-amyloid peptide aggregates (Aß_1–40_) or glycated beta-amyloid (Aβ)^86^. Aggregation of amyloid Aß peptides is a hallmark of AD and can cause cerebral amyloid angiopathy, microvascular changes and senile plaques^87^. On the other hand, glycated Aβ amyloid can bind to sugars forming glycation end products (AGEs) and generate the Aβ-AGE combination that is more active, that is, more toxic than non-glycated Aβ amyloids^88,89^. The Tucker3-QDA model showed all excitation/emission pairs in the region of the tyrosine residue present in Aβ amyloid (Fig. 7B,C)^86^. Only the 350 nm/469 nm pair can be attributed to both the coenzyme NADH region and the tyrosine residue^64,86^.

The parameters used to identify and classify the AD and healthy control groups were the fluorescence excitation and emission intensities. Since the models performed in this work were supervised models, the sample labels are initially known: samples with plasma from patients with AD and samples with plasma from healthy control group. Therefore, the model construction begins with machine training with samples further away from their groups' centers and then goes through the validation and testing stages, with samples with changes that most concern each class^34^. The models were built using their factors or components as a parameter. In this way, the model is able to predict the classes from the differences in the main fluorescence intensities or peaks of excitation and emission between the classes, but it cannot quantify this difference. However, by having the main excitation and emission peaks, future studies may confirm a new biomarker responsible for these signals or correlate with known biomarkers from plasma like Aβ_1–42_ and Aβ_1–40_, pTau forms, NfL and Glial fibrillary acidic protein (GFAP)^13,90^. Once the biomarker is known also be possible to try quantifying the excitation and emission intensity difference between the classes with specific analyses carried out for the specie or to quantify the levels of this biomolecule in the blood plasma of patients with AD and without the disease.

Despite previous studies describing the biological signatures associated with AD, there is a limitation in those that involve the fluorescence technique to determine specific biomolecule structures. Blood samples offer a complex matrix where fluorescent species and species with more than one fluorophore can coexist in the same structure. This can cause dubiousness when assigning signals to a specific analyte. In addition, the existing studies, in many cases, work on different sample matrices^64,86^, which can lead to signal displacement. Therefore, in this work, the assignments of fluorescence signals were only tentative. A suggestion for future studies is to carry out studies that confirm the relationship of the signals obtained here with biomolecules responsible for the biological signatures associated with AD. A starting point for new studies would be the excitation and emission wavelengths provided by loading profiles, which show the main wavelengths to differentiate the classes. Therefore, future studies should relate this information with structures of biomolecules that are known to be present in AD patients.

## Conclusions

The present study demonstrated a new tool for a fast, inexpensive and non-invasive blood-based method for diagnosing Alzheimer's disease. This is the first work using the EEM fluorescence spectroscopy and 2nd-order algorithms as an efficient screening tool for patients with Alzheimer's disease suspect. The PARAFAC-QDA and Tucker3-QDA models showed excellent results in the overall diagnostic performance as well as high discrimination power between the studied classes. Furthermore, the wavelengths found in the loadings' profiles are a starting point for future studies to discover or associate blood-based Alzheimer's disease biomarkers.

Our results are comparable and sometimes even better than conventional blood-based methods. When compared to Raman and IR spectroscopy, the EEM fluorescence spectroscopy is more sensitive and selective. In addition, EEM is less time-consuming than Raman spectroscopy, which has slow imaging by point scanning, and has cheaper instrumentation than ATR-FTIR (attenuated total reflectance Fourier transform infrared spectroscopy. Compared to current molecular blood-based tests, EEM fluorescence spectroscopy offers the advantage of an inexpensive, non-destructive and label-free diagnostic alternative. Future studies will work on developing this innovative molecular EMM fluorescence technique for AD diagnosis to provide a powerful diagnostic tool and expand its use for further clinical applications.

## Data Availability

The datasets used and/or analysed during the current study available from the corresponding author on reasonable request.
